# Design and synthesis of a novel nanocomposite based on magnetic dopamine nanoparticles for purification of α-amylase from the bovine milk

**DOI:** 10.1038/s41598-021-92919-0

**Published:** 2021-06-28

**Authors:** Reza Eivazzadeh-Keihan, Haniyeh Dogari, Farnoush Ahmadpour, Hooman Aghamirza Moghim Aliabadi, Fateme Radinekiyan, Ali Maleki, Leyla Saei Fard, Behnam Tahmasebi, Maryam Faraj Pour Mojdehi, Mohammad Mahdavi

**Affiliations:** 1 Catalysts and Organic Synthesis Research Laboratory, Department of Chemistry, Iran University of Science and Technology, 16846-13114 Tehran, Iran; 2 Protein Chemistry Laboratory, Department of Medical Biotechnology, Biotechnology Research Center, Pasteur Institute of Iran, Tehran, Iran; 3 Advanced Chemistry Studies Lab, Department of Chemistry, Toosi University of Technology, Tehran, K. N Iran; 4 School of Chemistry, College of Science, University of Tehran, Tehran, Iran; 5 Endocrinology and Metabolism Research Center, Endocrinology and Metabolism Clinical Sciences Institute, Tehran University of Medical Sciences, Tehran, Iran

**Keywords:** Chemical biology, Materials chemistry

## Abstract

In this paper, a novel nanocomposite based on magnetic nanoparticles decorated by dopamine were reported. Three modified magnetic nanocomposites by dopamine were offered with different type of linkers. The mentioned magnetic nanocomposites were applied to separate α-amylase protein from fresh bovine milk. All of the magnetic nanocomposites were characterized and investigated by using Fourier-transform infrared spectroscopy, energy-dispersive X-ray spectroscopy, field-emission scanning microscope, X-ray diffraction pattern, and vibrating-sample magnetometer analyses. To investigate the purifying application, sodium dodecyl sulfate polyacrylamide gel electrophoresis, one-dimensional isoelectric focusing gel electrophoresis, and alpha-amylase activity assay were employed. With paying attention to factors such as yield of purification and concentration of separated protein by each of magnetic nanocomposite, it could be concluded that the length of linkers played an important role in α-amylase protein separation. According to the results, the best separation and purification of α-amylase protein with 49.83% recovery and 40.11-fold purification efficiency was related to longest length linker, 1,4-butanediol diglycidyl ether, because of considerable conjugation with nanocomposite. Also, docking calculation has shown that the binding energy is − 1.697 kcal/mol and ΔG = − 6.844 kcal/mol which result that the interaction process between dopamine and α-amylase protein is spontaneous.

## Introduction

**Figure 1:**
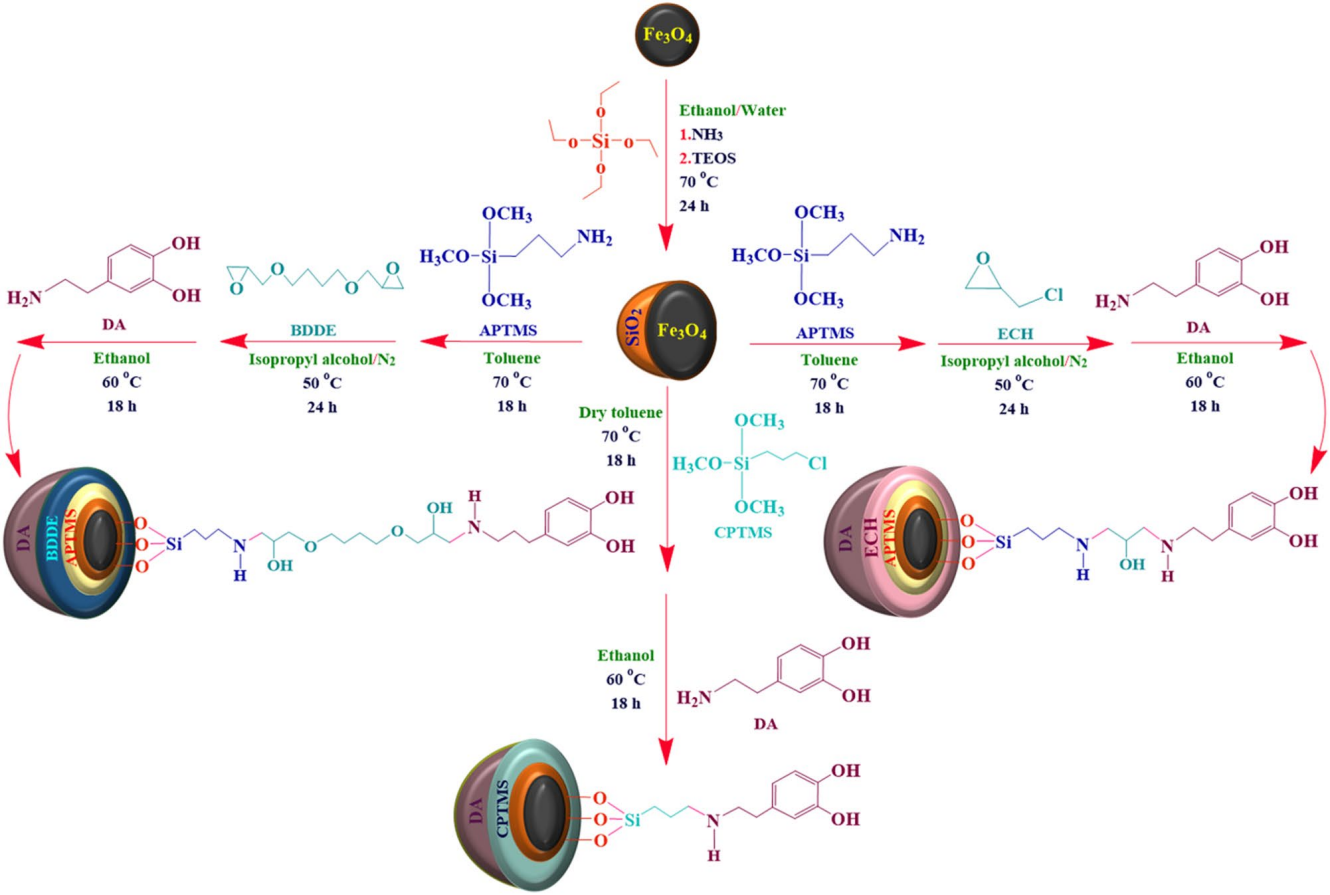
Schematic illustration of novel magnetic nanocomposites by modifying surface of Fe_3_O_4_ MNPs.

Proteins are one of the numerous classes of natural compounds which are constructed by repetitive amino acid groups^1^. In general, proteins are provided from animal and plant sources. In continue, some of plant sources are wheat, fruits, oats, rice, cereals, potatoes, and peas, and animal protein sources are meat, pork, beef, eggs, offal, and milk. According to the obtained information about proteins from animals and plants, most of the essential acid amines of human body are provided by animal more than plant sources^2^. Proteins with owning specific structures play an important physiological role in human body such as contractile and immune processes which are necessary and useful for the maintenance of muscle mass, and bone strength^3^. According to some special features of proteins like biocompatibility, and low toxicity, proteins are suitable candidates as protein-drug carriers in medical contexts; as well as, proteins can apply as an agent in drug delivery due to a bond between iron and proteins^4^. Apart from these descriptions, numerous methods exist to separate proteins with paying attention to their solubility, size, and other physical properties. Some knowledge about molecular mass, metal ion binding and solubility of proteins can help to choose a suitable method to separate them^5^. By considering previous research studies, chromatography methods are used to separate different proteins. Chromatography methods are categorized to different techniques including ion-exchange chromatography^6^, immobilized metal affinity chromatography^7^, and size exclusion chromatography^8^ to separate proteins. On the other side, electrophoresis methods are another procedures that can be applied to separate amino acids, and proteins^9^. Recently, magnetic nanocomposites have been highlighted as novel purification materials to apply in different contexts such as energy conversion^10,11^, catalysts^12,13^, enzyme and biomacromolecules separation^14^. Investigations of magnetic nanoparticles in biomedical fields such as tissue engineering^15,16^, detection of virus^17^, and cancer biomarkers^18^, are exclusively developed. As well as, scientists have focused in separation and adsorption of momentous and particular biomacromolecules such as DNA and proteins^19^ from complexes of their sources by magnetic-based nanocomposites. Separating and adsorbing of proteins can be the result of magnetic nanoparticles potency to interact with target biomacromolecule on their surface^20^. Controlling the size of nanoparticles by synthesized procedures is an important factor which must be considered for purification applications. The surface modification with organic or inorganic compounds such as metals^21^, and biomolecules^20^ make the nanocomposites able to create positive or negative charges on their surface to make interaction with proteins. Fe_3_O_4_ magnetic nanoparticles (Fe_3_O_4_ MNPs) with owning advanced features like high surface area and low toxicity are applied in separation of biomacromolecules^22^, hyperthermia of cancer therapy^23,24^, and catalytic agent in chemical reactions^25,26^. Fe_3_O_4_ MNPs are able to be modified like other magnetic nanoparticles by different kinds of chemical functional agent^27^. Based on previous studies about purification aspects, modified Fe_3_O_4_ MNPs are applied to separate heme proteins^28^, and α-amylase proteins^29^. In chemistry and biological contexts, enzymes have been used to start and continue reactions as a biological catalysts. These catalysts have more benefits in comparison to chemical catalysts. α-amylase (α-1,4-glucan-4-glucanohydrolase) proteins as biological catalysts received much attention in enzymatic reactions because of their abilities to hydrolyze starch^30^. This type of protein with three-dimensional structure is able to play an important role in food industry, textile, clinical, medicinal and chemistry fields^31^. In this research study, three novel magnetic nanocomposite based on modifying the surface of Fe_3_O_4_ MNPs are designed and synthesized with different shells such as tetraethyl orthosilicate (TEOS), (3-chloropropyl)trimethoxysilane (CPTMS), (3-aminopropyl)trimethoxysilane (APTMS), epichlorohydrin (ECH), 1,4-butanediol diglycidyl ether (BDDE), and dopamine (DA) (Fig. 1). These magnetic nanocomposites with different length and type of linkers are applied to separate α-amylase protein from fresh bovine milk. Obtained results from the separation of mentioned proteins are revealed that the length and type of linkers must be considered as important factors for protein purification. In addition to these descriptions, docking calculations have shown that the binding energy is − 1.697 (kcal/mol) and ΔG = − 6.844 (kcal/mol) which indicate that interaction process between dopamine (DA) and α-amylase protein is spontaneous.

## Experimental section

### Materials

All of the required chemical reagents and chemical solvents including hydrochloric acid (HCl, 37%. Mw = 36.46 g/mol), acetic acid (CH_3_COOH, ≥ 99%, Mw = 60.05 g/mol), iron (III) chloride hexahydrate salt (FeCl_3_·6H_2_O, 97%, Mw = 270.30 g/mol), iron (II) chloride tetrahydrate salt (FeCl_2_·4H_2_O, ≥ 99%, Mw = 198.81 g/mol), sodium hydroxide (NaOH, ≥ 97%, pellets, Mw = 40.00 g/mol), sodium chloride (NaCl, ≥ 99.5%, Mw = 58.44 g/mol), tetraethyl orthosilicate (Si(OC_2_H_5_)_4_, TEOS, 98%, Mw = 208.33 g/mol), (3-chloropropyl)trimethoxysilane (Cl(CH_2_)_3_Si(OCH_3_)_3_, CPTMS, ≥ 97%, Mw = 198.72 g/mol), tris (hydroxymethyl) aminomethane (C_4_H_11_NO_3_, tris base, ≥ 99.8%, Mw = 121.14 g/mol), (3-aminopropyl) trimethoxysilane (C_9_H_23_NO_3_Si, APTMS, 97%, Mw = 179.29 g/mol), epichlorohydrin (C_3_H_5_ClO, ≥ 99%, Mw = 92.52 g/mol), 1,4-butanediol diglycidyl ether (C_10_H_18_O_4_, BDDE, ≥ 95%, Mw = 202.25 g/mol), dopamine hydrochloride (C_8_H_11_NO_2_·HCl, Mw = 189.64 g/mol), 3,5-dinitrosalicylic acid (C_7_H_4_N_2_O_7_, DNS, Mw = 228.116 g/mol), ammonia solution (NH_4_OH. 25%, Mw = 35.05 g/mol), di*-*sodium hydrogen phosphate (Na_2_HPO_4_, Mw = 141.96 g/mol), sodium phosphate monobasic (NaH_2_PO_4,_ ≥ 99.5%, Mw = 119.98 g/mol), bradford solution (ultra-pure water, Coomassie brilliant blue g-250, ethanol 95%, orthophosphoric acid 85%), soluble starch ((C_6_H_10_O_5_)_n_), potassium sodium tartrate tetrahydrate (KNaC_4_H_4_O_6_.4H_2_O, 99%, Mw = 282.22 g/mol), tris(hydroxymethyl)aminomethane hydrochloride (tris–HCl, NH_2_C(CH_2_OH)_3_·HCl, ≥ 99.5%, Mw = 157.60 g/mol), glycine (C_2_H_5_NO_2_, ≥ 99%, Mw = 75.07 g/mol), 2-mercaptoethanol (C_2_H_6_OS, ≥ 99%, Mw = 78.13 g/mol), *N,N,N′,N′*-tetramethylethylenediamine (TEMED, C_6_H_16_N_2_, ~ 99%, Mw = 116.20 g/mol), acrylamide (C_3_H_5_NO, ≥ 99%, Mw = 71.08 g/mol), *N,N'*-methylenebis(acrylamide) (C_7_H_10_N_2_O_2_, 99%, Mw = 154.17 g/mol), sodium dodecyl sulfate (NaC_12_H_25_SO_4_, SDS, anionic, electrophoresis grade, Mw = 288.38 g/mol), ammonium persulfate ((NH_4_)_2_S_2_O_8_, APS, ≥ 98%, Mw = 228.20 g/mol), ethanol (C_2_H_5_OH, Mw = 46.07 g/mol), acetone (C_3_H_6_O, ≥ 99.5%, Mw = 58.08 g/mol), dry toluene (C_7_H_8_, anhydrous, 99.8%, Mw = 92.14 g/mol), and isopropyl alcohol (C_3_H_8_O, ≥ 99.7%, Mw = 60.10 g/mol) were procured in advance from international companies, Sigma-Aldrich, and Supelco. In addition to this, Amberlite IRN-150L and bromophenol blue (C_19_H_10_Br_4_O_5_S, Mw = 669.96 g/mol) were purchased from PlusOne company. Triton X-100 were purchased from Bio-Rad company. Also, pharmalytes for IEF (Ph 5–8, pH 7–9, and pH 3.5–10) were purchased from GE Healthcare company.

### Preparation of Fe_3_O_4_ MNPs

Magnetic precipitation of Fe_3_O_4_ MNPs was prepared by the prior reported method^24^. Initially, 2.91 g of FeCl_3_.6H_2_O, 1.33 g of FeCl_2_.4H_2_O (with a 2:1 ratio), and 150 mL of deionized water were mixed under N_2_ atmosphere and continuous heating conditions up to 70 °C. Then, 10 mL of ammonia was slowly added in 30 min. After the mentioned time, the reaction mixture was kept under the stirring condition at 70 °C for 3 h. After that, the external magnet was used to separate the obtained black precipitate. Then, it was washed with distilled water several times and dried at 70 °C for overnight (see supplementary information file, Fig. S1).

### Preparation of Fe_3_O_4_ MNPs coated by silica shell (Fe_3_O_4_@SiO_2_)

The surface functionalization of Fe_3_O_4_ MNPs with tetraethyl orthosilicate (TEOS) molecules was conducted by the following steps. First, 0.225 g of Fe_3_O_4_ powder was mixed with 25 mL of distilled water. This mixture was dispersed into ultrasonic bath for 20 min and then, 7.5 mL of ammonia was added to the mixture solution. After the mentioned time, 80 mL of ethanol was added to the mixture solution during 10 min. Next, 4 mL of TEOS was added and the reaction mixture was kept under the stirring condition at room temperature for 24 h. After the mentioned time, the obtained product was separated with an external magnet and washed by distilled water three times. Finally, it was dried at 70 °C for overnight (see supplementary information file, Fig. S2).

### Functionalization of Fe_3_O_4_@SiO_2_ MNPs using CPTMS molecules (Fe_3_O_4_@SiO_2_@CPTMS)

In this step, functionalization of Fe_3_O_4_@SiO_2_ MNPs was conducted by CPTMS molecules. First, 0.621 g of prepared Fe_3_O_4_@SiO_2_ precipitate was mixed with 60 mL of dry toluene. Then, the suspension solution was kept under the stirring condition and the temperature was raised up to 60 °C. Afterward, 1 mL of CPTMS was added to the suspension solution and stirred for 18 h at 60 °C. After the mentioned time, the precipitate was separated using an external magnet. In continue, the resulting product was washed with dry toluene for several times and dried at 70 °C for overnight (see supplementary information file, Fig. S3).

### Preparation of magnetic Fe_3_O_4_@SiO_2_@CPTMS@DA nanocomposite

To synthesis magnetic Fe_3_O_4_@SiO_2_@CPTMS@DA nanocomposite, first, the obtained product from previous synthesis step (Fe_3_O_4_@SiO_2_@CPTMS) was dispersed in 40 mL of ethanol. Then, 1.66 g of dopamine hydrochloride was added to the suspension solution and it was kept under the reflux condition for 18 h. After the mentioned time, the obtained product was separated by an external magnet and it was washed with distilled water and acetone to remove the unreacted dopamine. Ultimately, the prepared magnetic Fe_3_O_4_@SiO_2_@CPTMS@DA nanocomposite was dried at 60 °C for overnight (see supplementary information file, Fig. S4).

### Functionalization of Fe_3_O_4_@SiO_2_ MNPs using APTMS molecules (Fe_3_O_4_@SiO_2_@APTMS)

To functionalize Fe_3_O_4_@SiO_2_ MNPs using APTMS molecules, first, 0.621 g of Fe_3_O_4_@SiO_2_ powder was mixed with 60 mL of toluene. Suspension solution was stirred short time and heated up to 60 °C. Then, 1 mL of APTMS was drop wisely added to the suspension solution and kept under the stirring condition at constant temperature (60 °C) for 18 h. Afterwards, the obtained product was separated using an external magnet. The elution process was performed by dry toluene and it was dried using an oven at 70 °C (see supplementary information file, Fig. S5).

### Functionalization of Fe_3_O_4_@SiO_2_@APTMS MNPs using ECH molecules (Fe_3_O_4_@SiO_2_@APTMS@ECH)

Functionalization process of Fe_3_O_4_@SiO_2_@APTMS MNPs using ECH molecules was carried by following steps. First, 0.4 g of Fe_3_O_4_@SiO_2_@APTMS MNPs was mixed with 100 mL of isopropyl alcohol. Afterward, 5 mL of ECH was added to the mentioned mixture solution under the N_2_ atmosphere. The reaction was stirred for 24 h and then, the obtained product was separated with an external magnet and washed with isopropyl alcohol three times. Final product was dried on vacuum atmosphere at 50 °C (see supplementary information file, Fig. S6).

### Preparation of magnetic Fe_3_O_4_@SiO_2_@APTMS@ECH@DA nanocomposite

To coat DA shell on the obtained core–shell structure, in brief, 0.54 g of Fe_3_O_4_@SiO_2_@APTMS@ECH powder was dispersed in ethanol for 10 min. Afterwards, 1.75 g of DA was added to the mixture solution. Next, the mixture was refluxed for 18 h. Finally, the obtained product was separated using an external magnet and washed three times with distilled water to remove unreacted substances and the drying process was conducted at 60 °C for overnight (see supplementary information file, Fig. S7).

### Functionalization of Fe_3_O_4_@SiO_2_@APTMS MNPs using BDDE molecules (Fe_3_O_4_@SiO_2_@APTMS@BDDE)

To prepare Fe_3_O_4_@SiO_2_@APTMS@BDDE, first, 0.4 g of obtained Fe_3_O_4_@SiO_2_@APTMS precipitate was dispersed in 100 mL of isopropyl alcohol. After the complete dispersion, 5 mL of BDDE was added to the mixture solution under the N_2_ atmosphere and constant temperature (50 °C). Afterwards, the mixture solution was kept under the reflux condition for 24 h. After the mentioned time, the obtained product was separated using an external magnet and washed with isopropyl alcohol three times. The prepared magnetic product was kept on vacuum atmosphere at 50 °C to dry (see supplementary information file, Fig. S8).

### Preparation of magnetic Fe_3_O_4_@SiO_2_@APTMS@BDDE@DA nanocomposite

Magnetic Fe_3_O_4_@SiO_2_@APTMS@BDDE@DA nanocomposite was synthesized by coating DA shell on the surface of Fe_3_O_4_@SiO_2_@APTMS@BDDE MNPs. After, the complete dispersion of 0.54 g of Fe_3_O_4_@SiO_2_@APTMS@BDDE powder in ethanol (80 mL), 1.66 g of DA was added to the solution. The obtained mixture was kept under the reflux condition for 18 h. After the mentioned time, the magnetic product was separated using an external magnet and washed with ethanol several times. Finally, the obtained magnetic product was dried at 60 °C for overnight (see supplementary information file, Fig. S9).

### Fourier-transform infrared spectroscopy

The spectra were recorded using Fourier-transform infrared (FT-IR) spectrometer (Shimadzu FT-8400 s model, Japan) to characterize the formation of new functional groups in each synthesis step. 0.1–1.0% of each sample was well mixed into 200–250 mg of fine KBr powder for preparation of sample pellets. Considering the spectral resolution (4 cm^−1^) and a determined frequency range (400–4000 cm^−1^), each spectrum was taken at room temperature and the average number of scans was between 6 and 18^32^.

### Energy-dispersive X-ray spectroscopy

The elemental composition of sample was identified by energy-dispersive X-ray (EDX) device (SAMx model, France) with the accelerating voltage of 20 kV, 10 s live time, and using ultrathin window detector.

### Field-emission scanning microscopy

Using the field-emission scanning microscope (FE-SEM) (ZEISS-Sigma VP model, Germany), the Morphology, structure and size of samples were characterized, operating at a 15 kV. Each sample was mounted with double side carbon tape on stainless steel stub, and gold sputter-coating technique was performed (Agar Sputter Coater model, Agar Scientific, England). Besides, the images were taken with a determined scan rate (30 ns/pixel)^32^.

### X-ray diffraction pattern

The X-ray diffraction (XRD) pattern was recorded using Brucker X-ray diffractometer device (D8 Advanced Model, Germany). The device was equipped with Lynxeye detector (0D mode), and Cu-Kα radiation (λ = 0.154 nm, 40 kV, 40 mA). Considering a determined scan rate (0.2°/s), and angle scan was performed between the range of 5° ≤ 2*θ* ≤ 90°.

### Vibrating-sample magnetometer

Vibrating-sample magnetometer (VSM) was used to evaluate the saturation magnetization value (LBKFB model magnetic kavir, Iran). All the hysteresis loop curves were determined using an applied magnetic field from − 15,000 to + 15,000 Oe.

### Isolation and removal of cream and casein from bovine milk

First, fresh bovine milk was made from a local dairy and transferred to the lab. To separate the cream from the milk, the milk was centrifuged at 6000 rcf, for 15 min at 37 °C. Subsequently, to separate casein from the milk, the pH of obtained skim milk was reduced to 5 by hydrochloric acid solution on ice to precipitate casein. Then, the obtained solution was centrifuged at 6000 rcf, for 10 min at 10 °C^33^, and the pH of the supernatant was finally brought to 7 by sodium hydroxide solution. The obtained casein-free skim milk (CFSM) was used to continue the process.

### Isolation of α-amylase from the bovine milk by synthesized magnetic nanocomposites

α-Amylase isolation by synthesized magnetic nanocomposites, was performed according to the method of Farzi-Khajeh et al.^34,35^, preparing buffer A (50 mL of 0.05 M phosphate buffer containing 50 mM NaCl was made and its pH was adjusted to 7.8) and elution buffer B (50 mL of 0.05 M phosphate buffer containing 0.3 M NaCl was made and its pH was adjusted to 7.8). In the next step, 300 mg of each synthesized magnetic nanocomposite was mixed with 2.5 mL of buffer A and then 0.2 mL of CFSM was added and the solution was mechanically stirred for 30 min. Subsequently, each magnetic nanocomposite was separated using an external magnet from the suspension and washed three times by 2.5 mL of buffer A to remove unbound proteins. Following that, each isolated magnetic nanocomposite was stirred for 10 min with 1 mL of buffer B to separate α-amylase from their surface. Then, using a magnet, the solution containing α-amylase was removed from each nanocomposite and used for the rest of the steps.

### α-Amylase activity assay

α-Amylase activity assay was performed according to the method described by Zakowski et al. On this basis, 0.2 mL of CFSM was mixed with 0.3 mL of phosphate buffer (0.05 M) and 0.5 mL of starch solution (1% w/v) as a substrate, at 37 °C for 10 min. Then, to stop the reaction, 1 mL of DNS solution (1% w/v) was added and the solution was heated in a water bath for 5 min. Subsequently, 0.33 mL of potassium sodium tartrate solution (4% w/v) was added and cooled rapidly in ice. Finally, the absorbance of the resulting solution was measured at 540 nm and the maltose concentration was determined using a standard curve^5^.

### Measurement of total protein concentration

The protein amount of the samples was measured by biophotometer (Eppendorf) at 595 nm, according to the Bradford assay using bovine serum albumin (BSA) as standard^36^.

### Sodium dodecyl sulfate polyacrylamide gel electrophoresis (SDS-PAGE)

SDS-PAGE test was used for purity determination and molecular weight estimation of the purified enzyme. 12.5 μL of casein-free skim milk (CFSM), 20 μL of eluted samples from nanocomposites and 5 μL of prestained protein maker (BLUelf, GeneDirex) were separated by 10% resolving gel and 5% stacking gel which was run under 100 V for 90 min. The running buffer was tris–glycine (pH 8.3). After electrophoresis, the gel was fixed with fixation solution for 60 min, and then washed three times with ultra-pure water (UPW). Finally, the gel was stained with colloidal Coomassie brilliant blue (G*-*250) staining solution overnight, then de-stained with 1% v/v acetic acid solution for 1 h^37^.

### Interpret the result of SDS-PAGE analysis using quantity one software

The gel was scanned using a calibrated densitometer and analyzed by quantity one 1-D analysis software (Bio-Red, v4.6.3). As a result of this analysis, the relative quantity and peak density (peak OD) for each band were obtained and the α-amylase purification efficiency was calculated for all nanocomposites.

### One‐dimensional isoelectric focusing (1D-IEF) gel electrophoresis

To determine the purified α-amylase isoelectric point, 1D-IEF in slab gel was performed. This technique also confirmed the purity of the purified enzymes. According to the protocol, 10 mL of acrylamide gel solution containing a mixture of 5.5 g of urea (9.1 M final), 2 mL 10% v/v Triton X-100 (2% v/v final), 2 mL Milli-Q water, 1.35 mL acrylamide stock solution (30% w/v acrylamide, 1.6% w/v bisacrylamide; treated with amberlite), 0.2 mL ampholytes pH 5–8, 0.2 mL ampholytes pH 7–9 and 0.1 mL ampholytes pH 3.5–10 was prepared. Then, to initiate polymerization, 20 μL of APS (10% w/v) and 10 μL TEMED were added. Finally, the gel was poured using electrophoresis tools. 20 mM NaOH solution was used as anodic buffer and 20 mM H_3_PO_4_ solution was used as cathodic buffer. Subsequently, 3 μL of marker (IEF standards, Bio-Rad) and 8 μL of each sample were loaded into the wells and the gel was run under 350 V for 10 h. After complete the run, the gel was stained with colloidal Coomassie brilliant blue (R*-*250) staining solution overnight, then de-stained with acetic acid solution (1% v/v) for 1 h^38^.

## Result and discussion

Three magnetic nanocomposites were fabricated by modifying the surface of Fe_3_O_4_ MNPs with different linkers and shells (Fig. 1). The mentioned magnetic nanocomposites were characterized using various analysis methods (FT-IR, EDX, FE-SEM, XRD, VSM analyses). FT-IR analysis was used to indicate the formation of new functional groups, EDX analysis to characterize structural composition, XRD pattern to indicate the crystalline phase of Fe_3_O_4_ MNPs, and VSM analysis to evaluate the magnetic properties and saturation magnetization value; which are discussed, respectively. Apart from these analysis methods, to evaluate these magnetic nanocomposites for separation of α-amylase protein from fresh bovine milk, other analysis methods were applied such as SDS-PAGE analysis to determine weight molecular and purity of separated proteins, and 1D-IEF analysis to determine isoelectric point. Also, the molecular modeling and docking study were conducted too.

### Characterization of synthesized magnetic nanocomposites

#### FT-IR analysis

**Figure 2:**
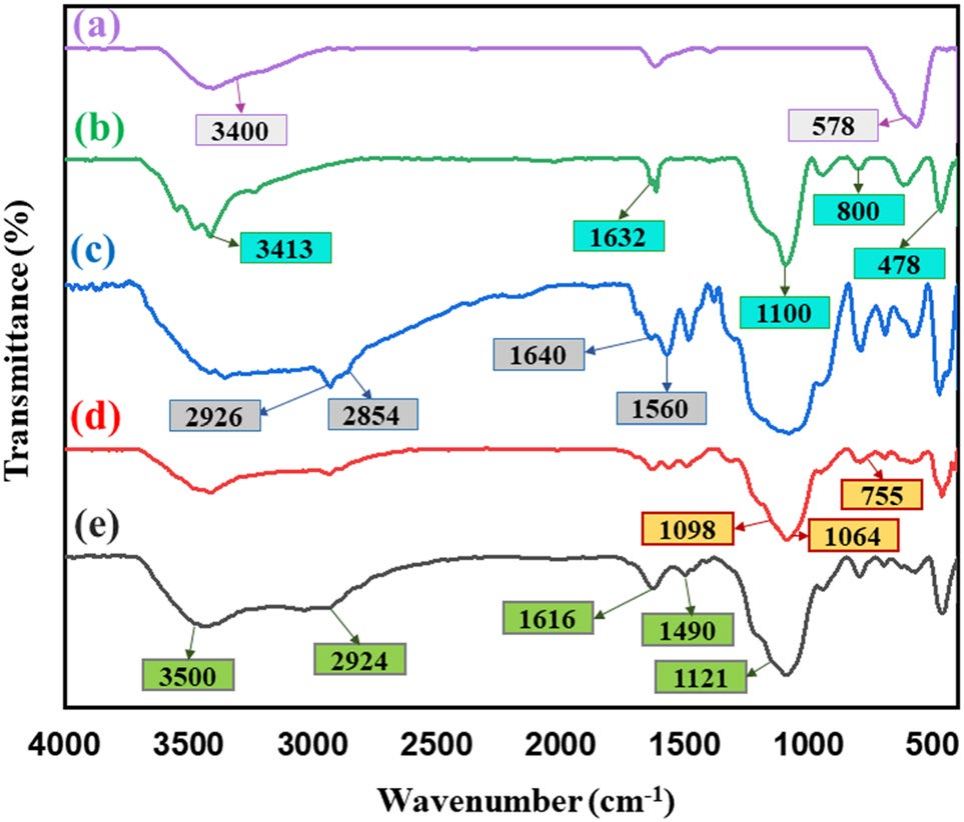
FT-IR spectra of (**a**) Fe_3_O_4_ MNPs, (**b**) Fe_3_O_4_@SiO_2_ MNPs, (**c**) Fe_3_O_4_@SiO_2_@APTMS MNPs, (**d**) Fe_3_O_4_@SiO_2_@APTMS@ECH MNPs, and (**e**) magnetic Fe_3_O_4_@SiO_2_@APTMS@ECH@DA nanocomposite.

As illustrated in FT-IR spectrum of Fe_3_O_4_ MNPs (Fig. 2a), an absorption band at around 578 cm^−1^ and a broadband at 3400 cm^−1^ are related to the stretching vibration modes of Fe–O and presented hydroxyl groups on the surface of nanoparticle^24,39,40^. Coating the silica shell on the surface of Fe_3_O_4_ core are accompanied by appearance of new functional groups (Fig. 2b). As could be seen, three absorption bands around 478 cm^−1^, 800 cm^−1^, and 1100 cm^−1^ can be attributed to the bending, symmetric, and asymmetric stretching vibration modes of Si–O–Si^41^. In addition to this, two absorption bands around 1632 cm^−1^ and 3200–3600 cm^−1^ (3413 cm^−1^) are assigned as stretching vibration mode of O–H, and O–H stretching vibration mode of Si–OH^42^. In continue, the presence of APTMS shell as second layer is characterized by observing new absorption bands. As indicated in Fig. 2c, two absorption bands around 1560 cm^−1^ and 1640 cm^−1^ are corresponded to C–N stretching and bending vibration modes of the amine group, respectively^43^. Appearing new absorption band at 2926 cm^−1^ and 2854 cm^−1^ can be attributed to the C–H stretching vibration of propyl group of linker^44^, as well as, a broad absorption band at region of 3300–3400 cm^−1^ can be related to the stretching vibration mode of N–H group of linker which has overlapped with Si–OH bond^45^. Following that, in Fig. 2d, small absorption band around 755 cm^−1^ can determine CH_2_–Cl functional group in the ECH structure^46^. Two broad absorption bands around 1098 cm^−1^ and 1064 cm^−1^ can be related to the epoxy group of ECH^47^. The FT-IR spectrum of magnetic Fe_3_O_4_@SiO_2_@APTMS@ECH@DA nanocomposite is indicated in Fig. 2e. The presence of three absorption bands around 1121 cm^−1^, 1490 cm^−1^, and 1616 cm^−1^ are assigned as aliphatic and aromatic C–H bending and N–H bonding vibration modes, respectively. Also, an absorption band around 2924 cm^−1^ can be ascribed to C–H stretching vibration mode of aromatic group^48^. Besides, a broad absorption band around 3350 cm^−1^ can be related to the presence of OH groups of dopamine^49^.

#### EDX analysis and FE-SEM imaging

**Figure 3:**
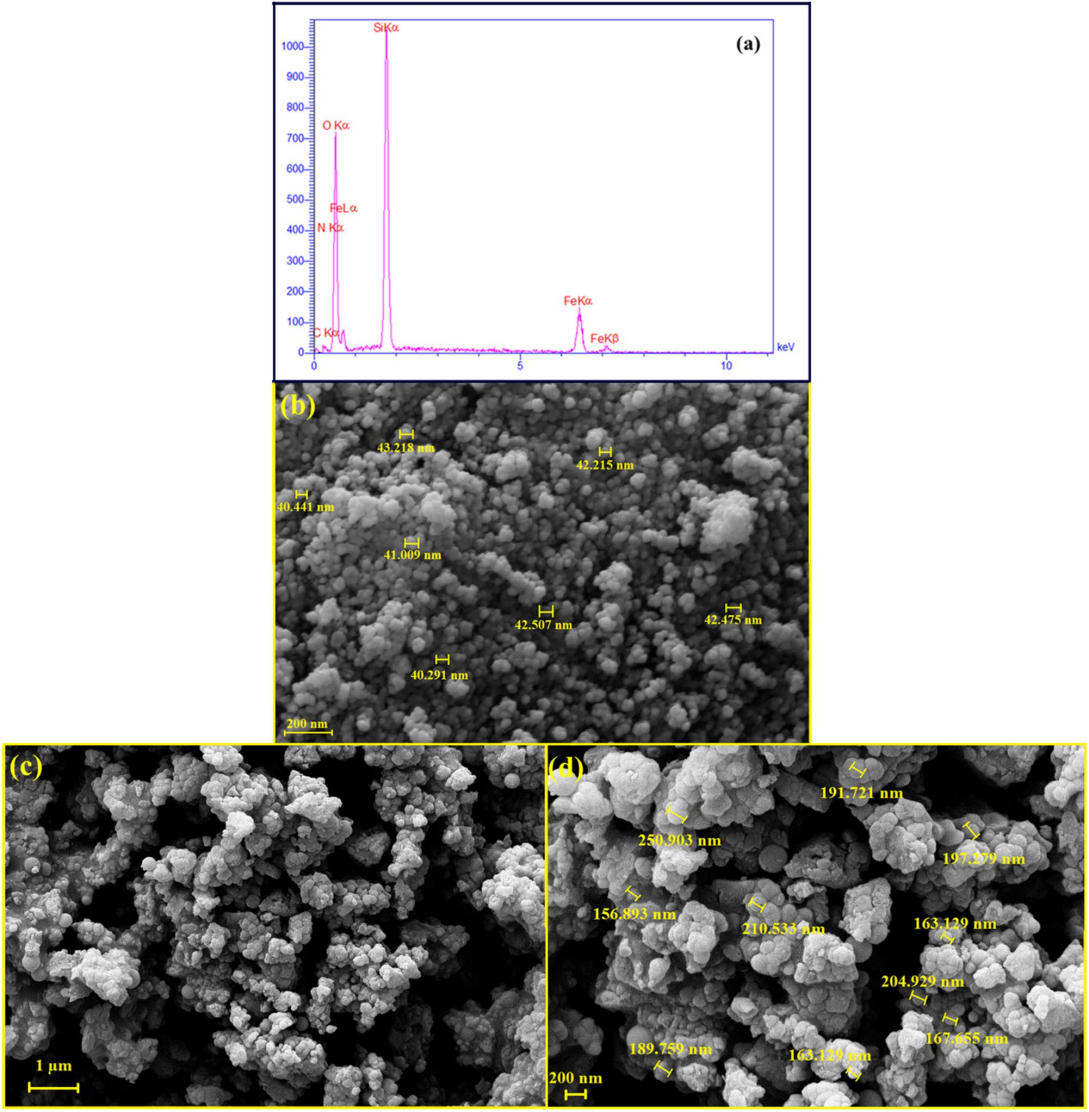
(**a**) EDX spectrum and FE-SEM images of (**b**) Fe_3_O_4_@SiO_2_ MNPs and (**c**,**d**) magnetic Fe_3_O_4_@SiO_2_@CPTMS@DA nanocomposites.

According to the EDX spectrum of magnetic Fe_3_O_4_@SiO_2_@CPTMS@DA nanocomposite (Fig. 3a), the presence of two iron peaks can be related to the magnetic Fe_3_O_4_ cores. Silicon, carbon, and oxygen peaks can confirm coating inorganic TEOS, CPTMS shells. Also, the presence of carbon and nitrogen peaks can be attributed to the coated DA structure. On the other side, given the FE-SEM imaging from magnetic Fe_3_O_4_@SiO_2_ MNPs and magnetic Fe_3_O_4_@SiO_2_@CPTMS@DA nanocomposite (Fig. 3b–d), the sphere morphology with almost uniform structure is observed. As well as, in comparison to the average size of Fe_3_O_4_@SiO_2_ MNPs (40–43 nm), the size of magnetic Fe_3_O_4_@SiO_2_@CPTMS@DA nanocomposite has increased from 155 to 250 nm; which is due to the surface functionalization process with different shells.

#### XRD pattern

**Figure 4:**
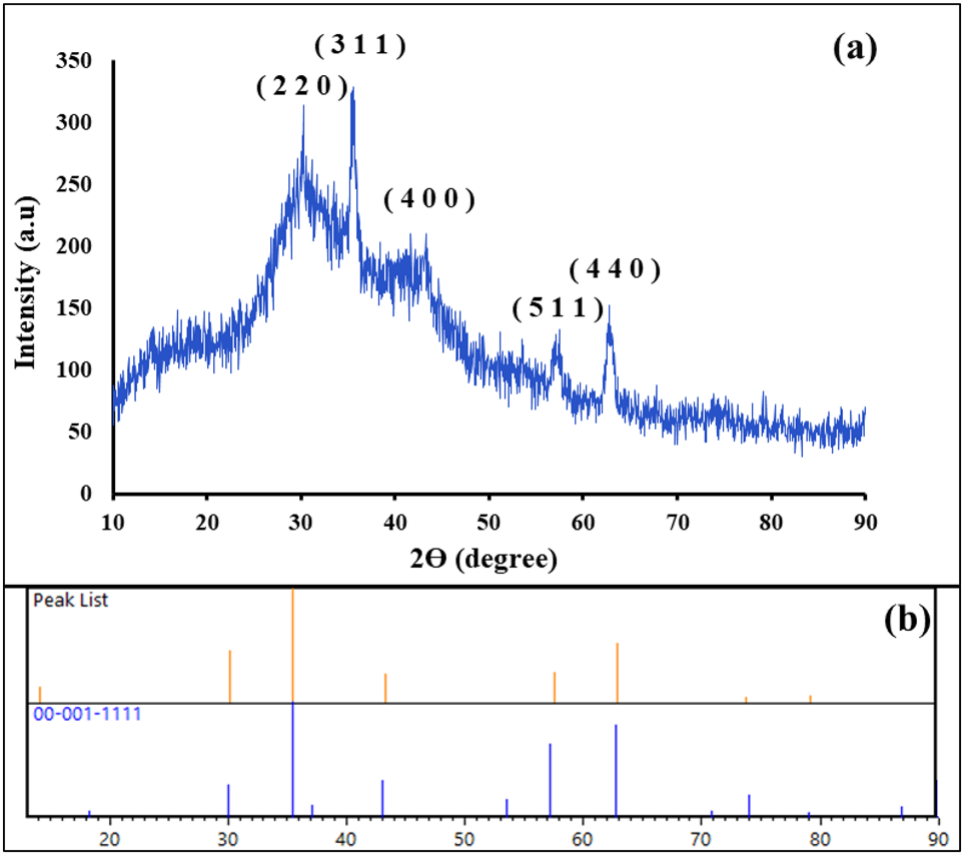
(**a**) XRD pattern of magnetic Fe_3_O_4_@SiO_2_@CPTMS@DA nanocomposite, and (**b**) reference of synthesized Fe_3_O_4_ MNPs in the structure of magnetic Fe_3_O_4_@SiO_2_@CPTMS@DA nanocomposite.

As can be observed, the XRD pattern of magnetic Fe_3_O_4_@SiO_2_@CPTMS@DA nanocomposite is indicated in Fig. 4a. The assigned peaks at the diffraction angles (2θ = 30.05, 35.45, 43.03, 57.20, 62.70) are related to the standard pattern of magnetic Fe_3_O_4_ cores (JCPDS card No. 00-001-1111) (Fig. 4b). Besides, the identified crystalline can be observed with their indices (2 2 0), (3 1 1), (4 0 0), (5 1 1), and (4 4 0)^50^.

#### VSM analysis

**Figure 5:**
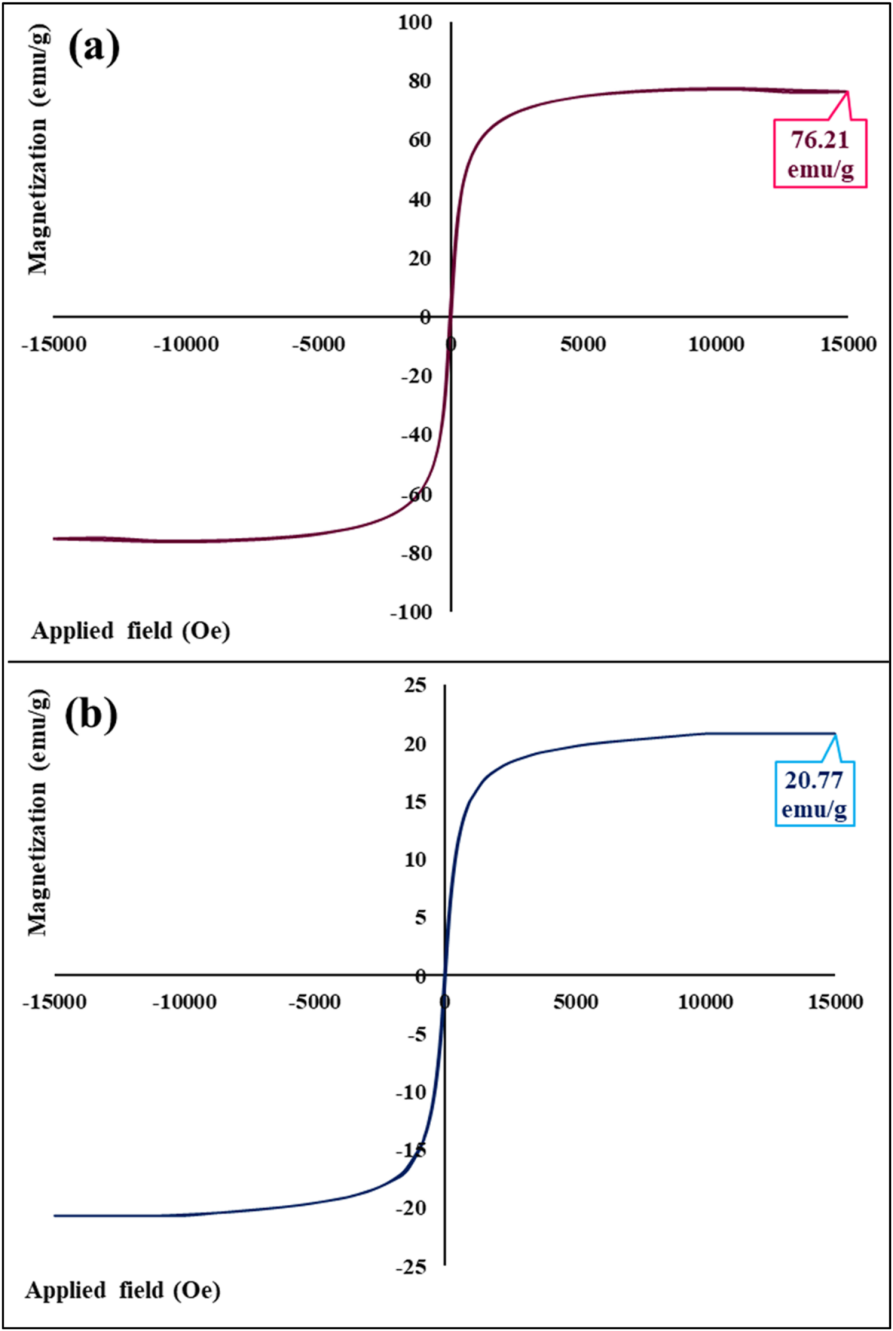
Hysteresis loop curves of (**a**) unfunctionalized Fe_3_O_4_ MNPs, and (**b**) magnetic Fe_3_O_4_@SiO_2_@CPTMS@DA nanocomposite.

In general, magnetic susceptibility and saturation magnetization value of magnetic-based nanostructures can be determined by vibrating-sample magnetometer analysis. It has been indicated that different factors including core size, shell thickness, interparticle and intraparticle interactions, and iron-group crystalline structure can impact on magnetic properties^51^. As could be seen in Fig. 5a,b, the saturation magnetization value of bare Fe_3_O_4_ MNPs before surface modifying is 76.20 emu/g (Fig. 5a); while this factor for magnetic Fe_3_O_4_@SiO_2_@CPTMS@DA nanocomposite has decreased to 20.77 emu/g (Fig. 5b). Considering reported literatures about surface modification of Fe_3_O_4_ MNPs and formation of various magnetic-based nanocomposites, it has been clarified that the contribution and immobilization process of non-magnetic shells on the surface of Fe_3_O_4_ cores can reduce their saturation magnetization value^52,53^. Therefore, it can be deduced that the observed reduction in saturation value of Fe_3_O_4_@SiO_2_@CPTMS@DA nanocomposite (20.77 emu/g) is related to the surface functionalization of Fe_3_O_4_ MNPs using inorganic and organic shells.

### Bio-application of synthesized magnetic nanocomposites

#### Evaluation and comparison of synthesized magnetic nanocomposites in purification of α-amylase

Magnetic Fe_3_O_4_@SiO_2_@CPTMS@DA (1), Fe_3_O_4_@SiO_2_@APTMS@ECH@DA (2), and Fe_3_O_4_@SiO_2_@APTMS@BDDE@DA (3) nanocomposites were synthesized by modifying the Fe_3_O_4_ surface using ligands immobilized through several different linker types with different lengths. These nanocomposites were applied for the purification of α-amylase; as well as the influence of the type and length of linkers was evaluated. The length of the linkers increased from Fe_3_O_4_@SiO_2_@CPTMS@DA (1) to Fe_3_O_4_@SiO_2_@APTMS@ECH@DA (2), and then to Fe_3_O_4_@SiO_2_@APTMS@BDDE@DA (3) nanocomposites, respectively. The results are summarized in Table 1 and indicate that the separation and purification of α-amylase from the sample matrix by all three nanocomposites performed well with high efficiency.

**Table 1:** Purification of α-amylase from the bovine milk by synthesized magnetic nanocomposites.

Fraction	Volume (ml)	Protein concentration (mg/ml)	Activity (mU/mL)	Specific activity^a^ (mU/mg protein)	Purification factor^b^	Yield^c^ (%)
Skim milk	500	15.29	594 ± 12	38.84	1	100
Casein-free skim milk	100	2.87	520 ± 12	181.18	4.66	87.54
(1)	1	0.16	186 ± 10	1162.5	29.93	31.31
(2)	1	0.17	208 ± 10	1223.52	31.50	35.01
(3)	1	0.19	296 ± 10	1557.89	40.11	49.83

^a^Specific activity [mU/mg] = enzyme concentration [mU/mL]/protein concentration [mg/mL], ^b^purification factor for (x) = specific activity of (x)/specific activity of starting material, ^c^yield = total enzyme activity of fraction (x)/total enzyme activity in starting material.

**Figure 6:**
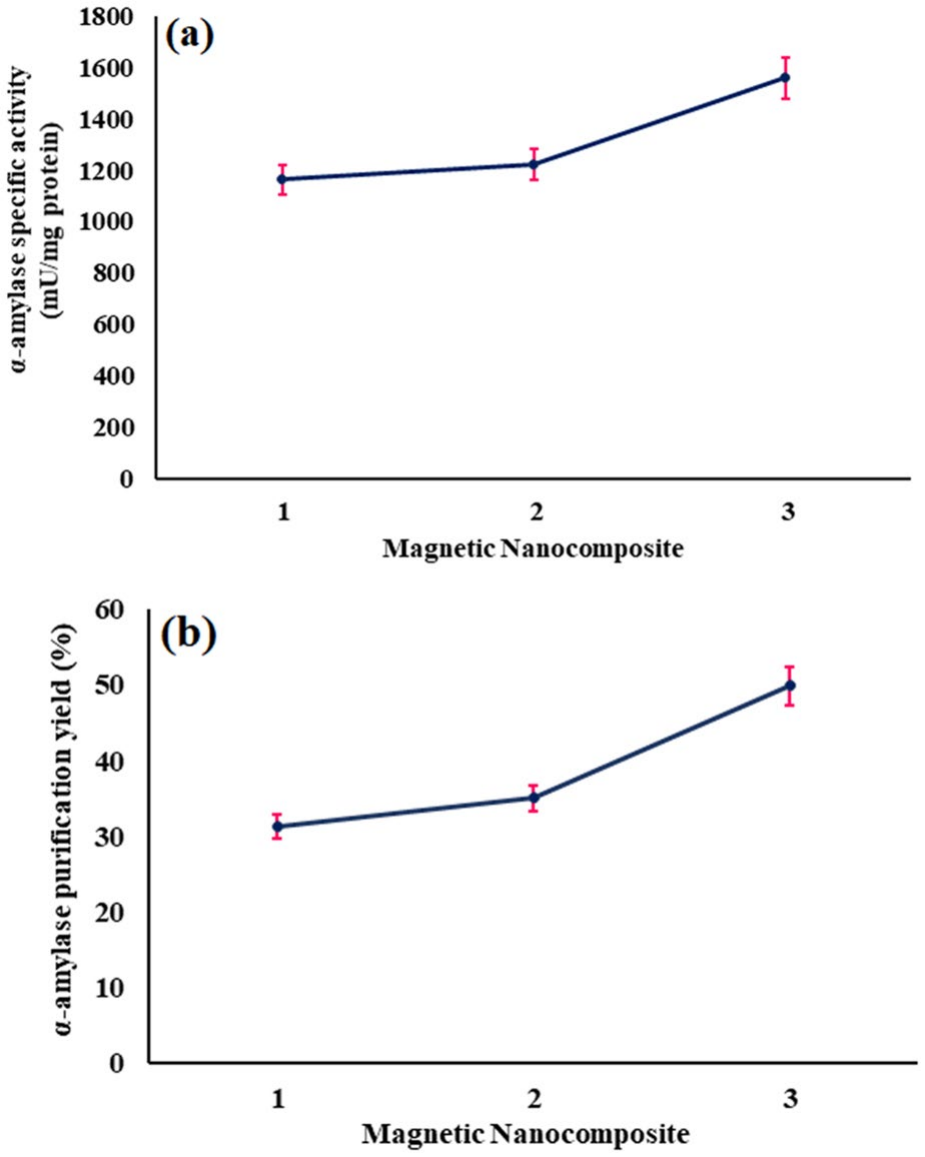
(**a**) Comparing the specific activity of α-amylase in magnetic Fe_3_O_4_@SiO_2_@CPTMS@DA (1), Fe_3_O_4_@SiO_2_@APTMS@ECH@DA (2), Fe_3_O_4_@SiO_2_@APTMS@BDDE@DA nanocomposites (3) (mU/mg protein), (**b**) comparing the purification yield of α-amylase by magnetic Fe_3_O_4_@SiO_2_@CPTMS@DA (1), Fe_3_O_4_@SiO_2_@APTMS@ECH@DA (2) and Fe_3_O_4_@SiO_2_@APTMS@BDDE@DA (3) nanocomposites based on specific activity.

Magnetic Fe_3_O_4_@SiO_2_@APTMS@BDDE@DA (3) nanocomposite, which has the longest linker, showed the highest efficiency in purification of α-amylase from bovine milk, which increased the specific activity of α-amylase by 40-fold; while, magnetic Fe_3_O_4_@SiO_2_@CPTMS@DA (1) and Fe_3_O_4_@SiO_2_@APTMS@ECH@DA (2) nanocomposites showed an approximately 30-fold increase. In explaining the cause, it can be said that the longer and more flexible and available linker for immobilizing the ligand in the structure of magnetic Fe_3_O_4_@SiO_2_@APTMS@BDDE@DA (3) nanocomposite, gives the ligand more access to α-amylase. Figure 6a compares the specific activity of α-amylase in purified by magnetic Fe_3_O_4_@SiO_2_@CPTMS@DA (1), Fe_3_O_4_@SiO_2_@APTMS@ECH@DA (2), and Fe_3_O_4_@SiO_2_@APTMS@BDDE@DA (3) nanocomposites. Also, Table 1 shows that the best and most efficient result for the separation and purification of α-amylase from the casein-free skim milk (CFSM), with 49.83% recovery and 40.11-fold purification measured based on specific activity, is related to Fe_3_O_4_@SiO_2_@APTMS@BDDE@DA (3) nanocomposite. Figure 6b compares the purification yield of α-amylase by magnetic Fe_3_O_4_@SiO_2_@CPTMS@DA (1), Fe_3_O_4_@SiO_2_@APTMS@ECH@DA (2) and Fe_3_O_4_@SiO_2_@APTMS@BDDE@DA (3) nanocomposites.

#### SDS-PAGE assay results

**Figure 7:**
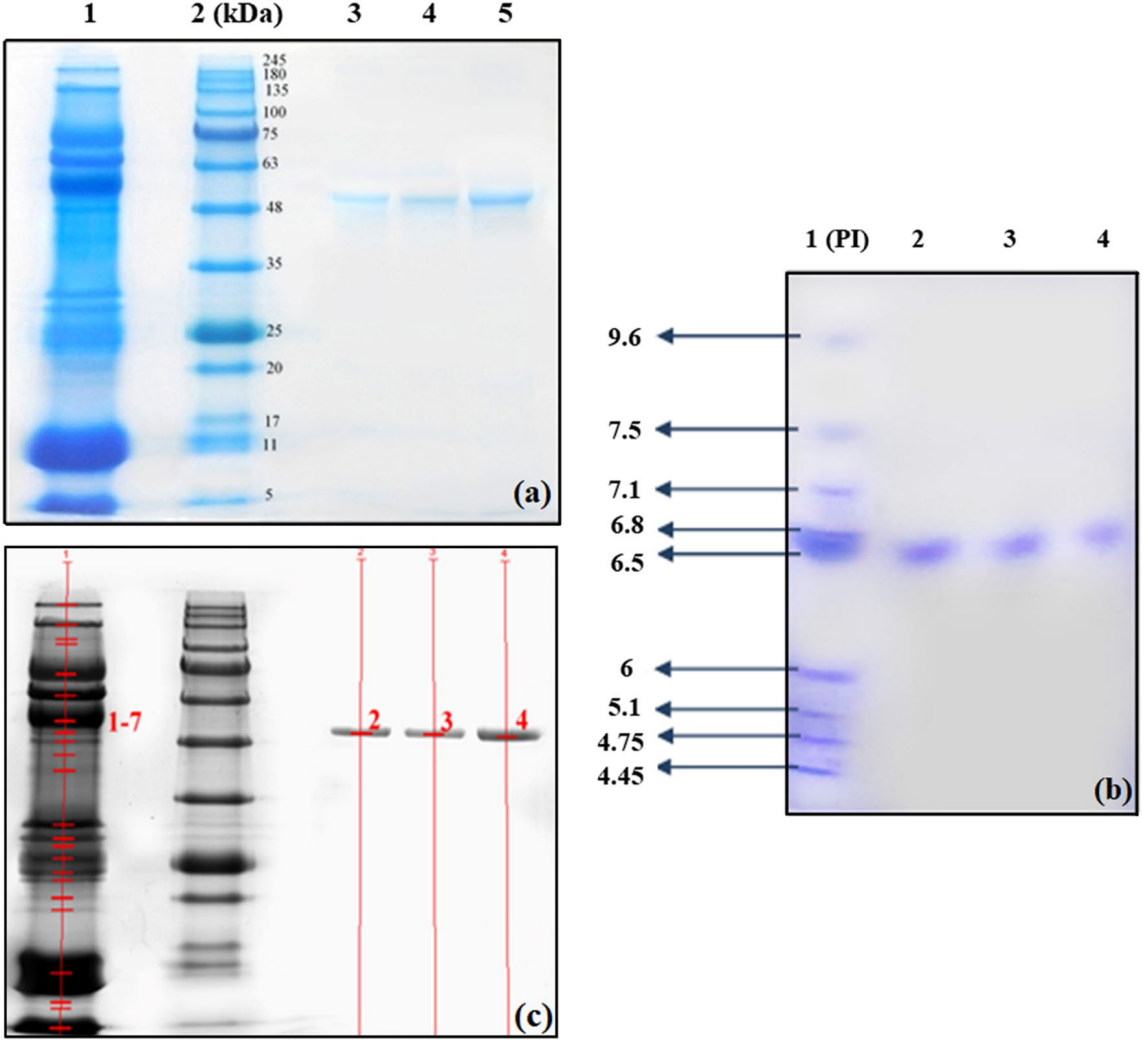
(**a**) SDS-PAGE analysis of: CFSM (lane 1), prestained protein ladder (BLUelf, cat no. PM008-0500) (lane 2), the eluted samples from: magnetic Fe_3_O_4_@SiO_2_@CPTMS@DA (1) (lane 3), Fe3O_4_@SiO_2_@APTMS@ECH@DA (2) (lane 4), Fe_3_O_4_@SiO_2_@APTMS@BDDE@DA (3) (lane 5) nanocomposites, (**b**) slab gel IEF analysis of: IEF standards marker (lane 1), the eluted samples from: magnetic Fe_3_O_4_@SiO_2_@APTMS@BDDE@DA (3) (lane 2) Fe_3_O_4_@SiO_2_@APTMS@ECH@DA (2) (lane 3), Fe_3_O_4_@SiO_2_@CPTMS@DA (1) (lane 4) nanocomposites, and (**c**) selected bands to analyze in quantity one software.

The results of the SDS-PAGE and single-band observation on the gel, without the presence of any significant impurities, indicate that all three magnetic nanocomposites bind specifically to α-amylase and do not bind to other proteins. As indicated in Fig. 7a, CFSM has many proteins that are observed in multiple bands in lane 1. After purification of α-amylase from CFSM, a major band of approximately 58 kDa molecular weight (MW) is obtained in lines 3, 4 and 5 on the gel. As can be seen in Fig. 7b, the α-amylase purified by the three magnetic nanocomposites including Fe_3_O_4_@SiO_2_@CPTMS@DA (1), Fe_3_O_4_@SiO_2_@APTMS@ECH@DA (2), and Fe_3_O_4_@SiO_2_@APTMS@BDDE@DA (3), has an isoelectric point (PI) of about 6.5–6.8. IEF analysis in addition to determining the pI of the isolated enzymes, confirms their SDS-PAGE results in terms of purity. It should be noted that in both Fig. 7a,b, the band derived from the α-amylase purified by magnetic Fe_3_O_4_@SiO_2_@APTMS@BDDE@DA (3) nanocomposite, is sharper than the other two bands, indicating that the purification of α-amylase from the bovine milk using magnetic Fe_3_O_4_@SiO_2_@APTMS@BDDE@DA (3) nanocomposite, is more effective and impressive. To complete the results, the purification efficiency of α-amylase by three magnetic Fe_3_O_4_@SiO_2_@CPTMS@DA (1), Fe_3_O_4_@SiO_2_@APTMS@ECH@DA (2) and Fe_3_O_4_@SiO_2_@APTMS@BDDE@DA (3) nanocomposites was calculated based on semi-quantitative analysis with quantity one software in addition to the specific activity calculations. Figure 7c shows the bands selected for analysis in the software (see supplementary information file, Figs. S10, S11).

Also, Table 2 shows the peak density (peak OD) and relative quantity of each band calculated by the software.

**Table 2:** Peak density and relative quantity in the desired bands.

Band	Peak OD	Relative Qty
1–7	0.96	9.7
2	0.49	100.0
3	0.50	100.0
4	0.65	100.0

Based on the results shown in Table 3, the purification efficiency of α-amylase was obtained for all three nanocomposites. As determined, magnetic Fe_3_O_4_@SiO_2_@APTMS@BDDE@DA (3) nanocomposite has the highest value with 67.7% purification efficiency (see supplementary information file, Fig. S12).

**Table 3:** Results of SDS-PAGE analysis based on semi-quantitative analysis with quantity one software.

Fraction	CFSM	(1)	(2)	(3)
The amount of sample injected into the well (μg)	57.4	3.2	3.4	3.8
The amount of α-amylase presented in the sample (μg)	5.56	2.83	2.89	3.76
α-Amylase purification efficiency (%)	100.00	50.89	51.97	67.70

#### Effect of incubation time on the adsorption of α-amylase to synthesized magnetic nanocomposites and their absorption capacity as nanocarrier

**Figure 8:**
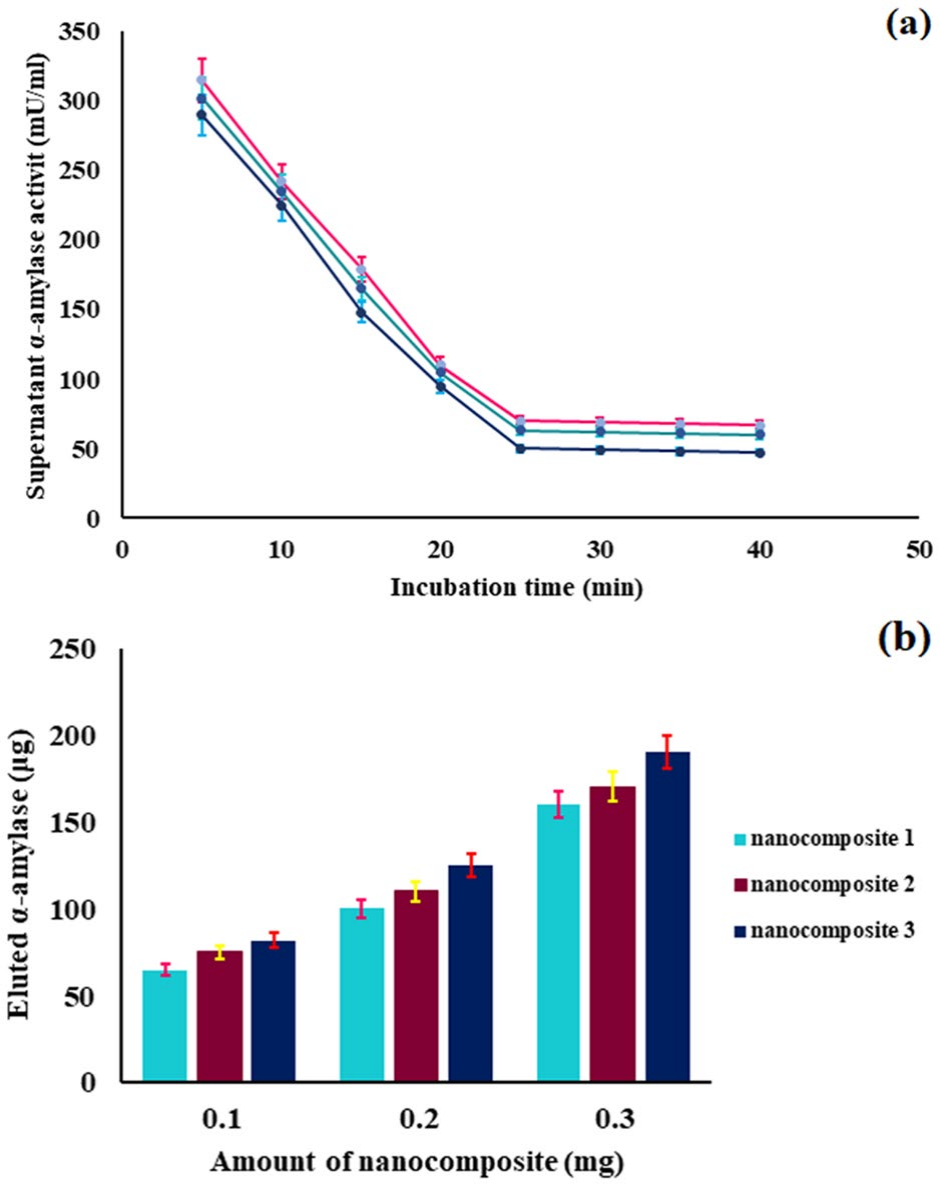
(**a**) Effect of incubation time on the adsorption of α-amylase, (**b**) absorption capacity of different amounts of magnetic nanocomposites.

300 mg of the nanocomposite was added to 2.5 mL of phosphate buffer and after dissolution, 0.2 mL of casein-free skim milk (CFSM) was added. The resulting mixture was incubated at room temperature for 5, 10, 15, 20, 25, 30, 35 and 40 min, after which the supernatant was separated from the nanocomposite by magnet and reacted with 0.5 mL of 1% w/v starch solution. At the end of each time period and after the supernatant was removed, α-amylase enzymatic activity was measured according to the procedure described in experimental section, the results of which are visible in Fig. 8a. According to the diagram, as the incubation time increased, α-amylase activity decreased, with no activity observed after 30 min. This is due to the increased adsorption of α-amylase to the surface of magnetic nanocomposites over time. 0.2 mL of casein-free skim milk (~ 0.57 mg protein) was stirred individually with 100, 200 and 300 mg of synthesized magnetic nanocomposites for 30 min at room temperature in phosphate buffer. The magnetic nanocomposites were then separated from the solution using a magnet and washed three times with phosphate buffer. Finally, α-amylase was eluted from the magnetic nanocomposites and total protein concentrations of the samples were measured. In addition, the adsorption capacity of all three magnetic nanocomposites was obtained by using following Eq. (1).

1 $${\text{Adsoption}}\;{\text{capacity}} = \left( {\frac{{{\text{An}}\;{\text{average}}\;{\text{amount}}\;{\text{of}}\;{\text{eluted}}\;\alpha {\text{ - amylase}}\;{\text{from}}\;{\text{nanocomposite}}\left( {\mu {\text{g}}} \right)}}{{{\text{An}}\;{\text{average}}\;{\text{amount}}\;{\text{of}}\;{\text{nanocomposite}}\;({\text{mg}})}}} \right)$$

The results of which are visible in Table 4 and Fig. 8b. Accordingly, the capacity of the magnetic Fe_3_O_4_@SiO_2_@APTMS@BDDE@DA nanocomposite  is higher than the others, which can be due to the longer linker on the surface of the nanocomposite, resulting in improved BDDE ligand access to α-amylase.

**Table 4:** Adsorption capacity of synthesized magnetic nanocomposites.

Nanocomposite	Adsorption capacity
(1)	0.541 ± 0.019
(2)	0.591 ± 0.025
(3)	0.661 ± 0.028

#### α-Amylase desorption from synthesized magnetic nanocomposites

To investigate the effect of salt concentration on α-amylase desorption, the absorbed α-amylase was desorbed in 0.05 M phosphate buffer with PH 7.8 containing NaCl with a concentration range of 0.05–0.3 M. According to the results, as the salt concentration increases, the amount of eluted α-amylase increases, indicating that the electrostatic forces play an important role in the interaction of α-amylase to immobilized ligands on the surface of nanocomposites. This is illustrated in Fig. S11, where it is observed that the highest amount of the adsorbed α-amylase can be eluted at 0.25 M NaOH, however, for the magnetic Fe_3_O_4_@SiO_2_@APTMS@BDDE@DA nanocomposite, this concentration is equal to 0.3 M (see supplementary information file, Fig. S13).

#### Comparison

In 2017, Farzi-Khajeh et al., synthesized nanocomposites with different components and lengths and used them to separate α-amylase from bovine milk. The highest purification efficiency (calculated by specific activity) with these nanocomposites was 49.66% and the maximum adsorption capacity was 0.466 ± 0.023 μg protein (α-amylase) per mg^34^. While in this present study, the highest purification efficiency, is 49.83% based on specific activity and 67.70% based on semi-quantitative analysis by quantity one software and as well, the maximum absorption capacity has improved to 0.661 ± 0.028.

### Molecular modeling and docking studies

This study has conducted using the reliable docking software Glide included in the package Maestro 10.2. The full protein sequence of amylase (pdb:1ppi) was retrieved from protein data bank (PDB) and prepared by protein preparation wizard module embedded in Maestro 2017. In other word, water, other molecules and ions were removed from the PDB structure. Then optimization and minimization were performed on amylase by force field OPLS-3. The binding site was determined based on 1ppi. Glide was used the preparation box set to 25 × 25 × 25 Å and centered at the point with − 10, 45 and 25. The 3D structure of DA is drawn with Gauss View 5, then were optimized using the density function theory (DFT) method^54^. Using Beck's three-parameter hybrid function and the Lee–Yang–Parr nonlocal correlation function (B3LYP) and 6–31 + G^*^ basis set^55,56^. Finally, the docking experiment was performed using the Maestro algorithm by Glide^57^. Docking calculation has shown that the binding energy is − 1.697 (kcal/mol). Free energy calculation was performed using the MM-GBSA method^58^. ΔG = − 6.844 (kcal/mol) shows that this process is spontaneous. In this calculation, the number of hydrogen bond acceptors is 2.5, the number of hydrogen bond donors is 4 and molecular weight is 153.180 (g/mol), subsequently, in this calculation Lipinski’s rule is not violated^59^. Also, Predicted central nervous system activity for this structure is inactive (see supplementary information file, Fig. S14)^60^.

## Conclusions

Nowadays, new and high-tech procedures have made a big progress in separation and purification methods. Magnetic nanocomposites are one of those procedures which are employed widely in separation technologies especially in purification of important macromolecules like proteins. In current research, a unique magnetic nanocomposite which decorated by dopamine biomolecules, was designed and synthesized. Three different types of linker  with different length were used to decorate magnetic nanoparticles by dopamine. The mentioned nanocomposite was evaluated in separation of α-amylase protein from fresh bovine milk. Structure and morphology of nanocomposite was characterized and investigated by using FT-IR, EDX, FE-SEM, XRD, and VSM analyses. Sodium dodecyl sulfate polyacrylamide gel electrophoresis, one-dimensional isoelectric focusing gel electrophoresis and alpha-amylase activity assay were used to investigate yield of α-amylase protein purification. After evaluation of obtained results, it was clearly concluded that the length of linkers played an important role in α-amylase protein separation. The best separation and purification of α-amylase protein with 49.83% recovery and 40.11-fold purification efficiency was related to longest length linker, 1,4-butanediol diglycidyl ether, because of considerable conjugation with nanocomposite. Docking calculation has shown that the binding energy is − 1.697 kcal/mol and ΔG = − 6.844 kcal/mol which result that interaction process between DA and α-amylase protein is spontaneous.

## Supplementary Information

Supplementary Information 1.
