# Supplementary material for: Design and synthesis of a novel nanocomposite based on magnetic dopamine nanoparticles for purification of α-amylase from the bovine milk
Source: Sci Rep. 2021 Jun 28;11:13428. doi: 10.1038/s41598-021-92919-0 (PMC8239001; doi:10.1038/s41598-021-92919-0)
Supplement: Supplementary file 1 — Supplementary Information 1. [file 41598_2021_92919_MOESM1_ESM.pdf]

Supplementary information

Design and synthesis of a novel nanocomposite based on magnetic dopamine nanoparticles for purification of α-amylase from the bovine milk

Reza Eivazzadeh-Keihan[a], Haniyeh Dogari[a], Farnoush Ahmadpour[a], Hooman Aghamirza Moghim Aliabadi[b,c], Fateme Radinekiyan[a], Ali Maleki[a]*, Leyla Saei Fard[a], Behnam Tahmasebi[d], Maryam Faraj Pour Mojdehi[a], Mohammad Mahdavi[e]

[a]*Catalysts and Organic Synthesis Research Laboratory, Department of Chemistry, Iran University of Science and Technology, Tehran 16846-13114, Iran*

[b]*Protein Chemistry Laboratory, Department of Medical Biotechnology, Biotechnology Research Center, Pasteur Institute of Iran, Tehran, Iran*

[c]*Advanced Chemistry Studies Lab, Department of Chemistry, K. N. Toosi University of Technology, Tehran, Iran*

[d]*School of Chemistry, College of Science, University of Tehran, Tehran, Iran*

[e]*Endocrinology and Metabolism Research Center, Endocrinology and Metabolism Clinical Sciences Institute, Tehran University of Medical Sciences, Tehran, Iran*

**Corresponding author. E-mail: maleki@iust.ac.ir; Fax: +98-21-73021584; Tel: +98-21-73228313.*

Entry	Subject	Page
2	Fig. S1. Synthesis process of Fe_3O_4 MNPs	S3
4	Fig. S2. Synthesis process of functionalized Fe_3O_4@SiO_2 MNPs	S4
5	Fig. S3. Synthesis process of Fe_3O_4@SiO_2@CPTMS MNPs	S5
6	Fig. S4. Synthesis process of magnetic Fe_3O_4@SiO_2@CPTMS@DA nanocomposite	S6
7	Fig. S5. Functionalization of Fe_3O_4@SiO_2 MNPs using APTMS molecule	S7
8	Fig. S6. Functionalization of Fe_3O_4@SiO_2@APTMS MNPs using ECH molecules	S8
9	Fig. S7. Synthesis process of magnetic Fe_3O_4@SiO_2@APTMS@ECH@DA nanocomposite	S9
10	Fig. S8. Functionalization of Fe_3O_4@SiO_2@APTMS MNPs using BDDE molecules	S10
11	Fig. S9. Synthesis process of magnetic Fe_3O_4@SiO_2@APTMS@BDDE@DA nanocomposite	S11
12	Fig. S10. The uncropped image of 1D-IEF slab gel	S12
13	Fig. S11. Uncropped image of SDS-PAGE gel	S13
14	Fig. S12. Comparison of purification efficiency percentage of α-amylase by magnetic Fe_3O_4@SiO_2@CPTMS@DA (1), Fe_3O_4@SiO_2@APTMS@ECH@DA (2), and Fe_3O_4@SiO_2@APTMS@BDDE@DA (3) nanocomposites based on semi-quantitative analysis with quantity one software	S14
15	Fig. S13. Effects of NaCl concentration on the α-amylase desorption from synthesized magnetic nanocomposites	S15
16	Fig. S14. Schematic illustration of molecular modeling of α-amylase separation	S16

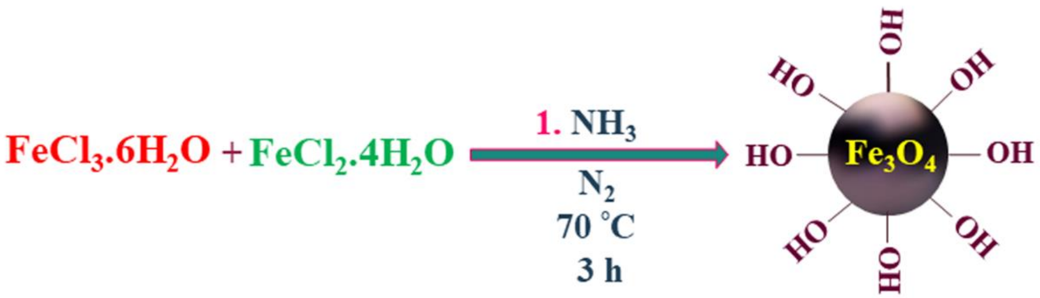

Fig. S1. Synthesis process of Fe_3O_4 MNPs.

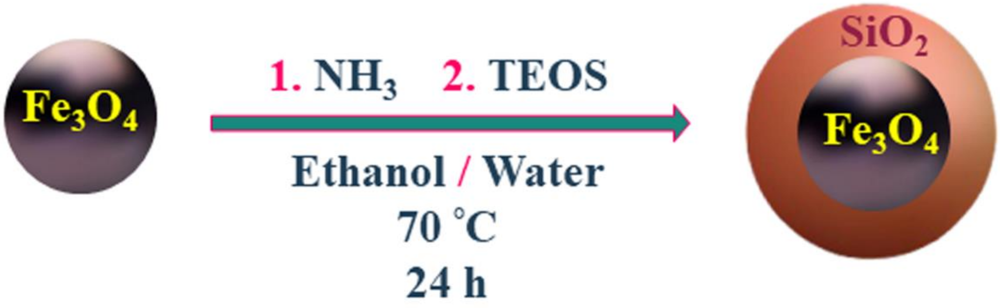

Fig. S2. Synthesis process of functionalized Fe_3O_4@SiO_2 MNPs.

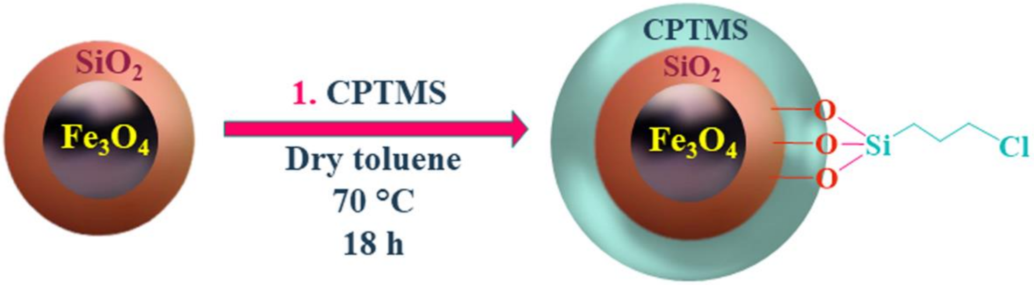

Fig. S3. Synthesis process of Fe_3O_4@SiO_2@CPTMS MNPs.

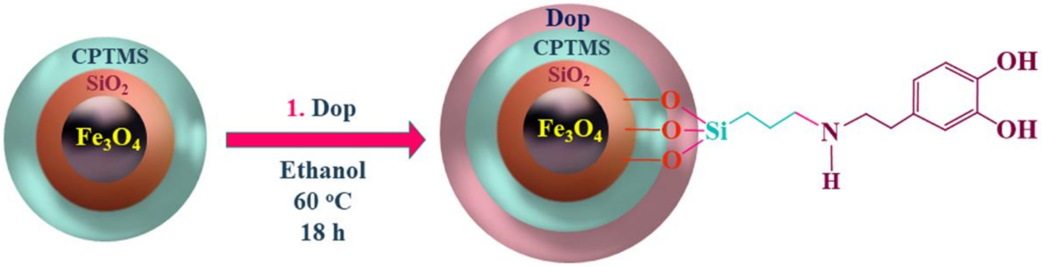

Fig. S4. Synthesis process of magnetic Fe_3O_4@SiO_2@CPTMS@DA nanocomposite.

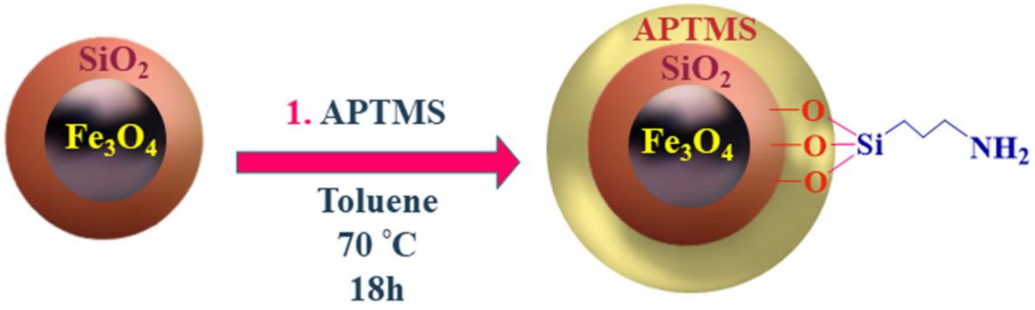

Fig. S5. Functionalization of Fe_3O_4@SiO_2 MNPs using APTMS molecule.

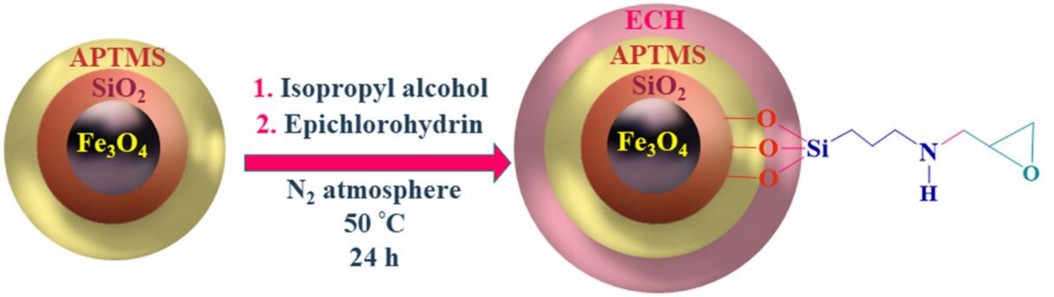

Fig. S6. Functionalization of Fe_3O_4@SiO_2@APTMS MNPs using ECH molecules.

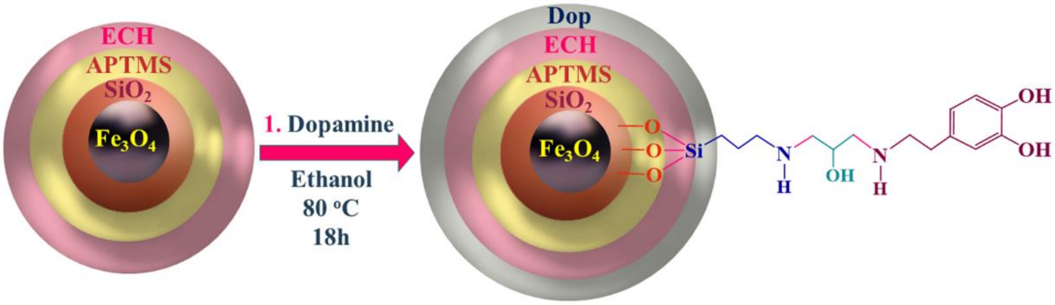

Fig. S7. Synthesis process of magnetic Fe_3O_4@SiO_2@APTMS@ECH@DA nanocomposite.

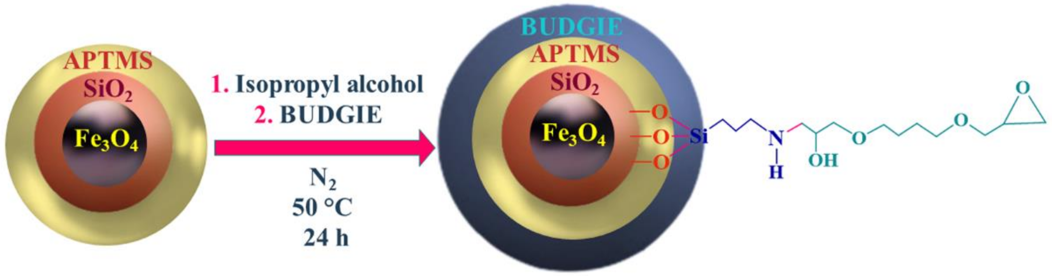

Fig. S8. Functionalization of Fe_3O_4@SiO_2@APTMS MNPs using BDDE molecules.

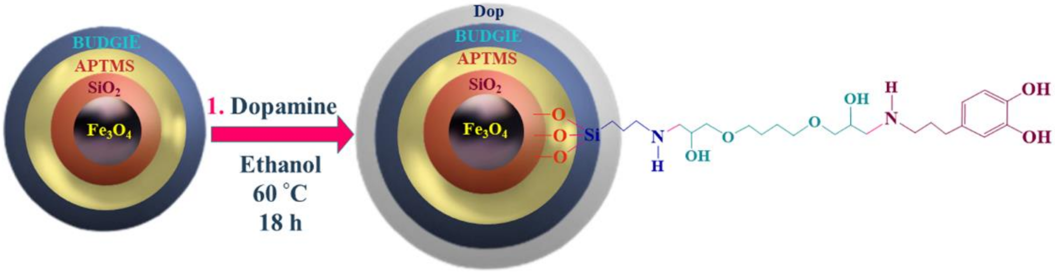

Fig. S9. Synthesis process of magnetic Fe_3O_4@SiO_2@APTMS@BDDE@DA nanocomposite.

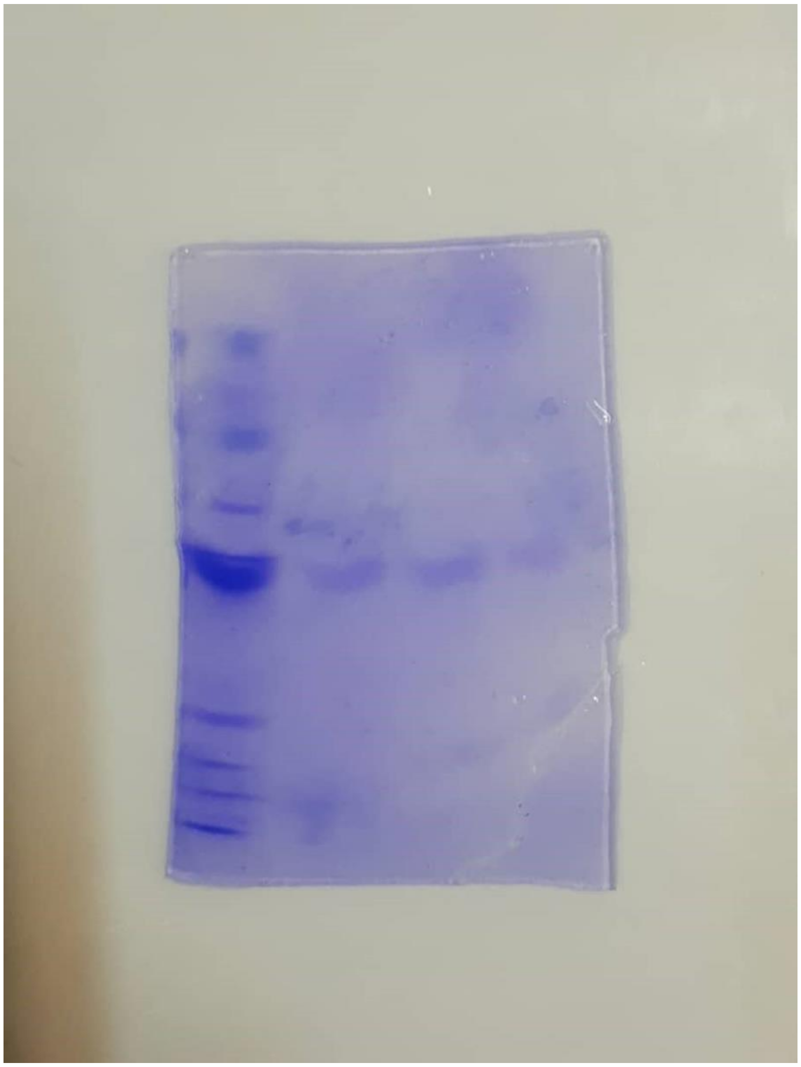

Fig. S10. Uncropped image of 1D-IEF slab gel.

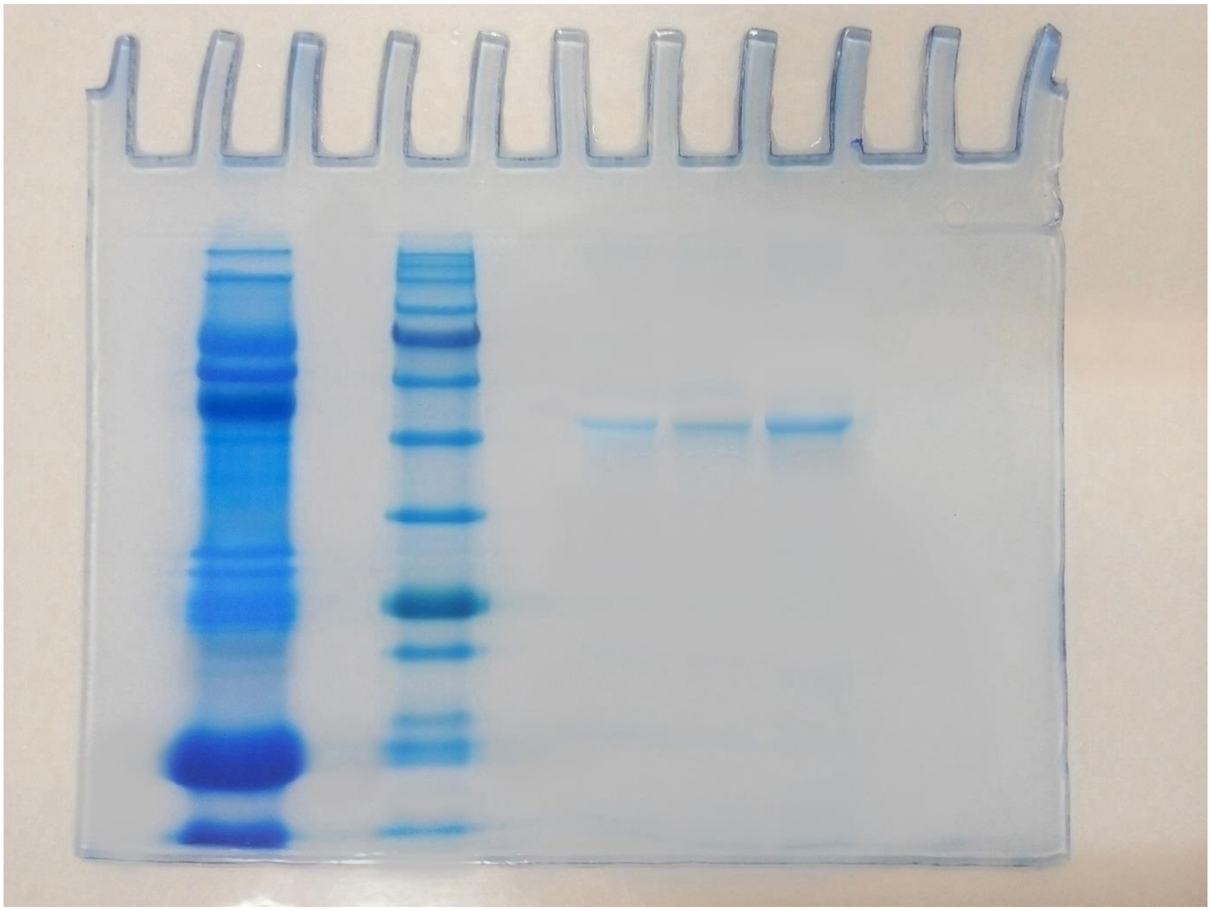

Fig. S11. Uncropped image of SDS-PAGE gel.

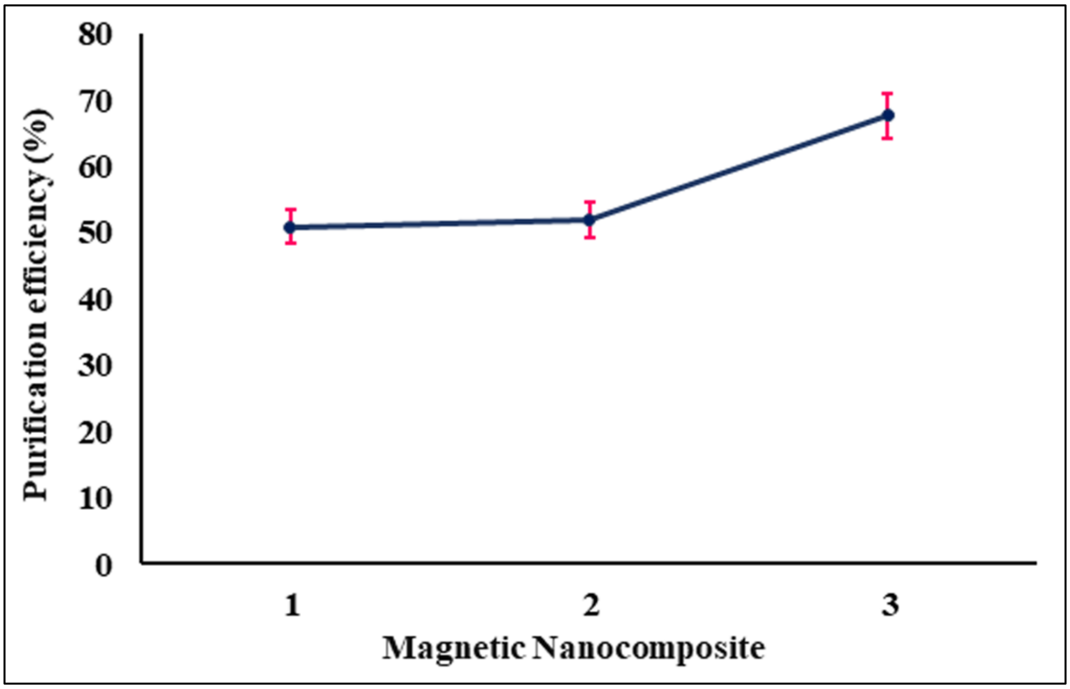

Fig. S12. Comparison of purification efficiency percentage of α-amylase by magnetic Fe_3O_4@SiO_2@CPTMS@DA (1), Fe_3O_4@SiO_2@APTMS@ECH@DA (2), and Fe_3O_4@SiO_2@APTMS@BDDE@DA (3) nanocomposites based on semi-quantitative analysis with quantity one software.

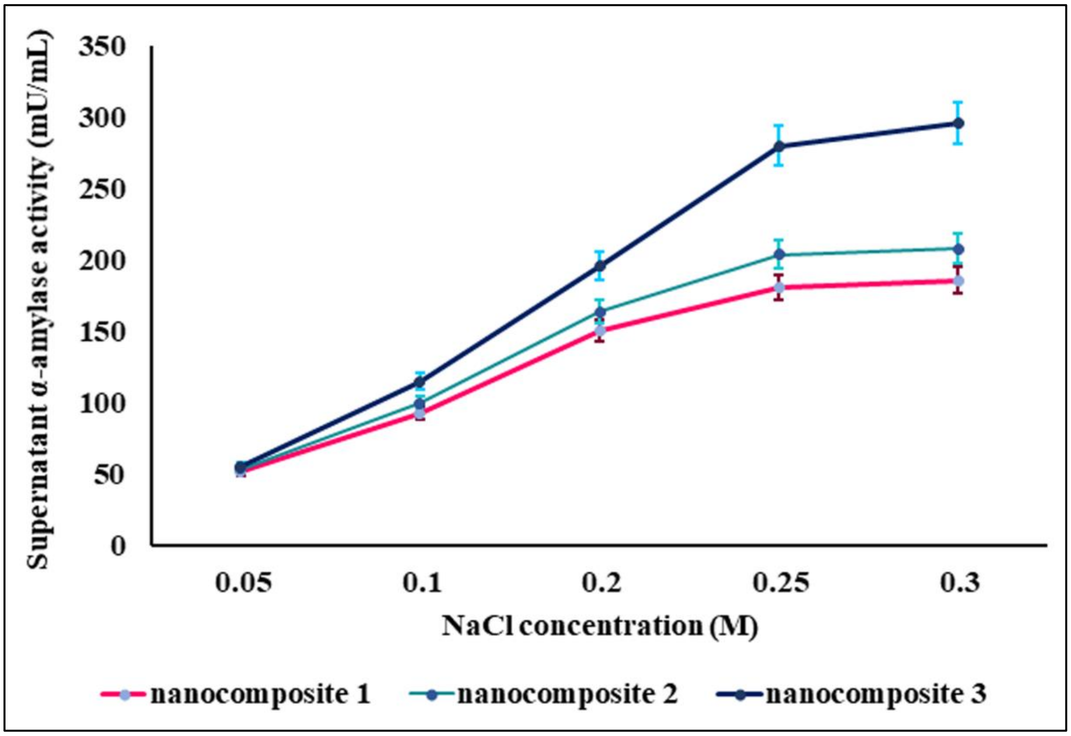

Fig. S13. Effects of NaCl concentration on the α-amylase desorption from synthesized magnetic nanocomposites.

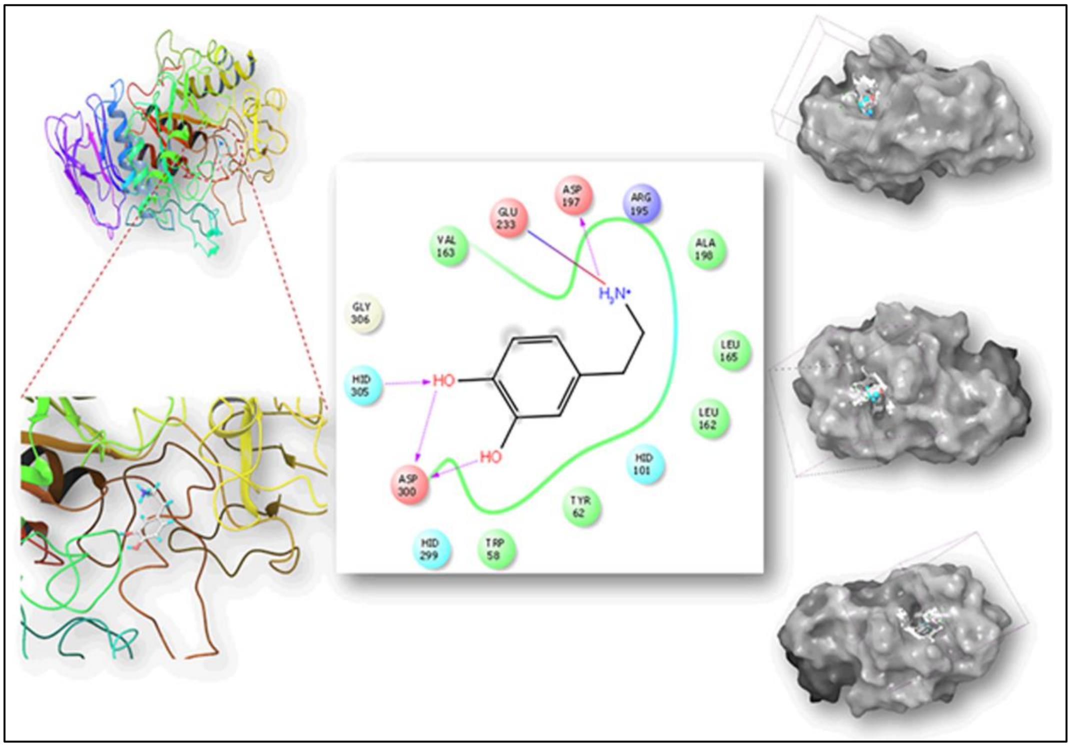

Fig. S14. Schematic illustration of molecular modeling of α-amylase separation.
